# Ten years of weekly epidemiological teleconference (EpiLag) – an effective and time-efficient tool for infectious disease event information, Germany, 2009–2018

**DOI:** 10.1017/S095026882100073X

**Published:** 2021-04-12

**Authors:** Astrid Milde-Busch, Nadine Zeitlmann, Inge Mücke, Andreas Gilsdorf, Ute Rexroth, Maria an der Heiden, the EpiLag-Working Group, Viviane Bremer, Florian Burckhardt, Hermann Claus, Michaela Diercke, Helmut Fickenscher, Sebastian Haller, Carina Helmeke, Stefan Hell, Anja Hauri, Katharina Katz, Carmen Kellner, Carolin Lampe, Felix Lange, Dorothea Matysiak-Klose, Anika Meinen, Sophie-Susann Merbecks, Elke Mertens, Anita Plenge-Bönig, Julia Schilling, Sabine Schroeder, Claudia Siffczyk, Doreen Staat, Christiane Wagner-Wiening, Dirk Werber

**Affiliations:** Robert Koch Institute, Berlin, Germany

**Keywords:** Communication, events, infectious diseases, surveillance, tool

## Abstract

In 2009, the Robert Koch Institute (RKI) and the 16 German federal state public health authorities (PHAs) established a weekly epidemiological teleconference (EpiLag) to discuss infectious disease (ID) events and foster horizontal and vertical information exchange. We present the procedure, discussed ID topics and evaluation results of EpiLag after 10 years. We analysed attendance, duration of EpiLag and the frequency of reported events. Participants (RKI and state PHA) were surveyed regarding their satisfaction with logistics, contents and usefulness of EpiLag (Likert scales). Between 2009 and 2018, RKI hosted 484 EpiLag conferences with a mean duration of 25 min (range: 4–60) and high participation (range: 9–16; mean: 15 PHAs). Overall, 2975 ID events (39% international, 9% national and 52% subnational) were presented (mean: 6.1 per EpiLag), most frequently on measles (18%), salmonellosis (8%) and influenza (5%). All responding participants (14/16 PHAs and 9/9 at RKI) were satisfied with the EpiLag's organization and minutes and deemed EpiLag useful for an overview and information distribution on ID events relevant to Germany. EpiLag is time efficient, easily applicable and useful for a low-threshold event communication. It supports PHAs in crises and strengthens the network of surveillance stakeholders. We recommend its implementation to other countries or sectors.

## Introduction

Germany is a federal republic consisting of 16 federal states and 401 districts [1]. In the area of infectious disease (ID) surveillance, public health authorities (PHAs) at three administrative levels (national, state and local) carry out tasks on the legal basis of the German Infection Protection Act (‘*Infektionsschutzgesetz*’; IfSG), the Decision No. 1082/2013/EU and the International Health Regulations (IHR, 2005).

**Fig. 1.**
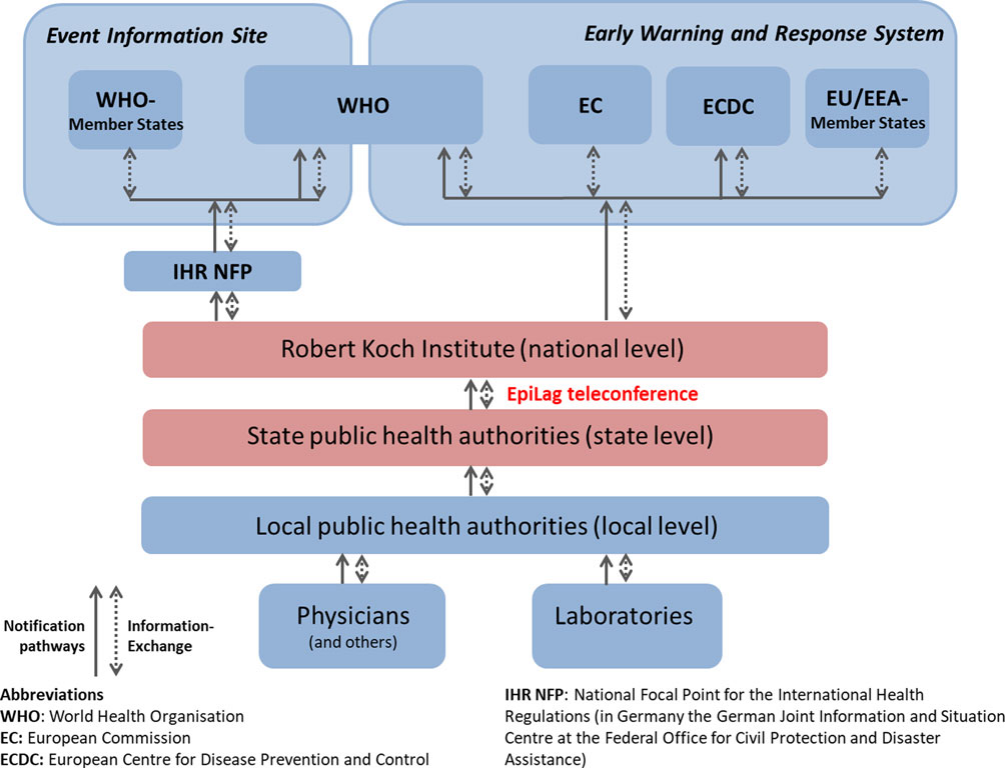
The German mandatory surveillance system for infectious diseases.

According to the IfSG, specific ID and pathogens have to be notified mandatorily within the ID surveillance system. Most of them are reported by laboratories and physicians to the local PHA. The latter usually receive the case notification on paper (e.g. via fax), enter the respective information in their surveillance software and transmit it electronically through the respective state PHA to the Robert Koch Institute (RKI) (Fig. 1). RKI develops and maintains a software that can be used for this purpose at all levels. In the software, each administrative entity (local, federal state, national level) has access to their respective data. However, cases belonging to the same event (regardless of their geographical origin within Germany) can be linked in the software via outbreak identifiers [2, 3].

The local PHA's responsibility lies in verifying notifications, detecting and investigating ID cases and events, and implementing control measures. The state PHA may support investigations at local level and analyses federal-state wide data. RKI analyses the nation-wide notification data (including application of regular outbreak algorithms), establishes surveillance case definitions and has access to official international early warning systems and communication platforms such as the Early Warning and Response System (EWRS) of the EU, the WHO's Event Information Site (EIS) and the Epidemic Intelligence Information System (EPIS) from the European Centre for Disease Prevention and Control (ECDC).

Surveillance stakeholders exchange routinely on ID notification data and events through the notification software and weekly bulletins. Furthermore, RKI hosts physical (face-to-face) meetings with the state PHA three times a year.

Nevertheless, as regular timely information exchange between the 16 state PHAs (horizontally) and the RKI (vertically) on relevant events remained challenging, a weekly epidemiological telephone conference (‘*Epidemiologische Lagekonferenz*’; EpiLag) was established in 2009.

Its goal was to provide a communication platform for the 16 state PHAs and the RKI to discuss relevant ID events on international, national and subnational level with low communication barriers. The facilitation of vertical dissemination of information to all levels of the public health sector was equally an objective. Furthermore, the EpiLag aims to foster horizontal exchange (and thereby to early detect new events and linked cases), to support the harmonisation of control measures and to discuss best practices between the federal states.

In order to fulfil the stated objectives, a simple and effective format is needed, that allows achieving high participation, is able to obtain, document and disseminate information from and to the participants reliably and rapidly, and that is robust and flexible in times of crises.

As no reports of similar procedures or tools could be found in literature, this manuscript aims to present an overview of the applied procedure (in order to inspire other countries), discussed ID events and evaluation results of 10 years of EpiLag (January 2009–December 2018).

## EpiLag procedures

The EpiLag is conducted as a weekly teleconference and takes place every Tuesday, except on or following national bank holidays. Participation is voluntary and comprises representatives of the Department for Infectious Disease Epidemiology at RKI and the epidemiologists responsible for ID surveillance and control at the 16 state PHAs.

During the EpiLag, relevant ID events, notification software-related issues and ‘other topics’ (e.g. legal or organisational issues) are discussed. ID events are subcategorized into international, national and subnational events. While the RKI reports mainly international and national events, the state PHA generally inform about subnational events in their geographical area. International events, events occurring mainly outside or beyond Germany, are identified from screening EWRS, EPIS, EIS, the Communicable Disease Threat Reports (CDTR) issued by the ECDC and ProMED-Mail. Other official sources might be used as necessary.

## Criteria for reporting

**Fig. 2.**
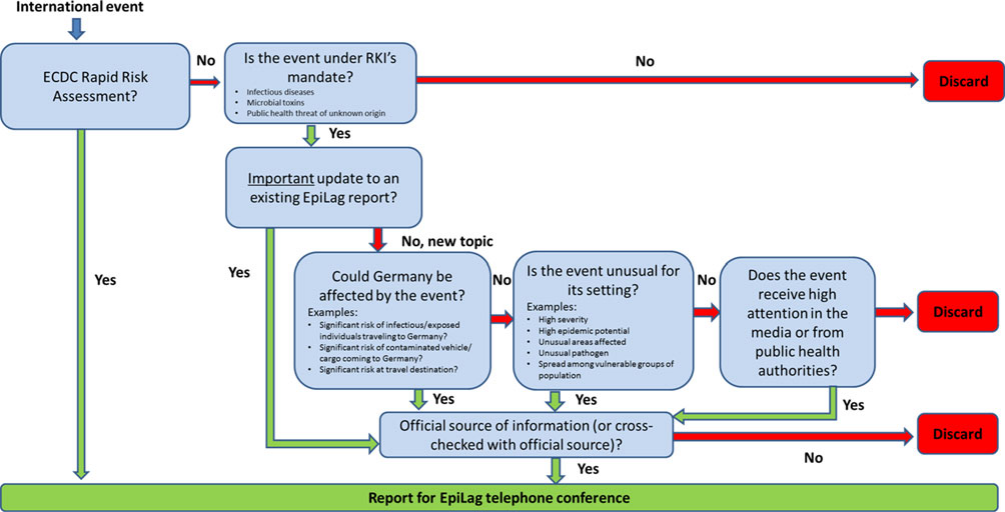
Selection criteria for international events to be reported during the EpiLag, Germany, 2009–2018.

The selection criteria for reporting international events can be retrieved from Figure 2.

RKI reports every Rapid Risk Assessment (RRA) published by ECDC and all other international events from above named sources that could affect Germany, are unusual or receive high attention within the public health community or in the media.

Epidemiologists from the disease-specific units at the Department for Infectious Disease Epidemiology at RKI select national events for EpiLag, for example, if a relevant outbreak affects several federal states and/or result in a need for recommendations to the subnational levels. This might occur when, for example, clusters of Listeriosis cases with certain sequence types are brought to the attention of RKI by the national reference laboratory: when the potential vehicle is unknown, EpiLag can be a means to ask local PHA to interview associated cases with trawling questionnaires. Another example might be that the RKI identifies an increased number of sporadic cases of a specific phage type of *Salmonella:* The RKI may use the EpiLag then to recommend local PHA to send isolates of newly-notified cases for typing to national reference laboratories in order to measure the extent of the outbreak.

Events from state PHA are selected by epidemiologists in the PHA; there is no standardized set of criteria for event selection. This is due to the constitutional independence of the federal states but also to keep the reporting threshold as low as possible. Reporting is voluntary and does not require prior information to the organizers. State PHA may, for example, choose to report events affecting more than one federal state during the EpiLag in order to raise awareness, so the linkage of cases in multiple federal states can be undertaken. If one federal state, for example, encounters a measles outbreak associated with a mass event (e.g. music festival) or a travel group to another federal state (e.g. a school trip), EpiLag is used to inform PHA authorities of other federal states of this event so they can alert their participants of these events to take public health action and link those cases through one outbreak identifier in the software. But also, smaller local outbreaks or single infections are reported if deemed unusual or with a potentially serious public health impact.

## Organisation and weekly procedure

**Fig. 3.**
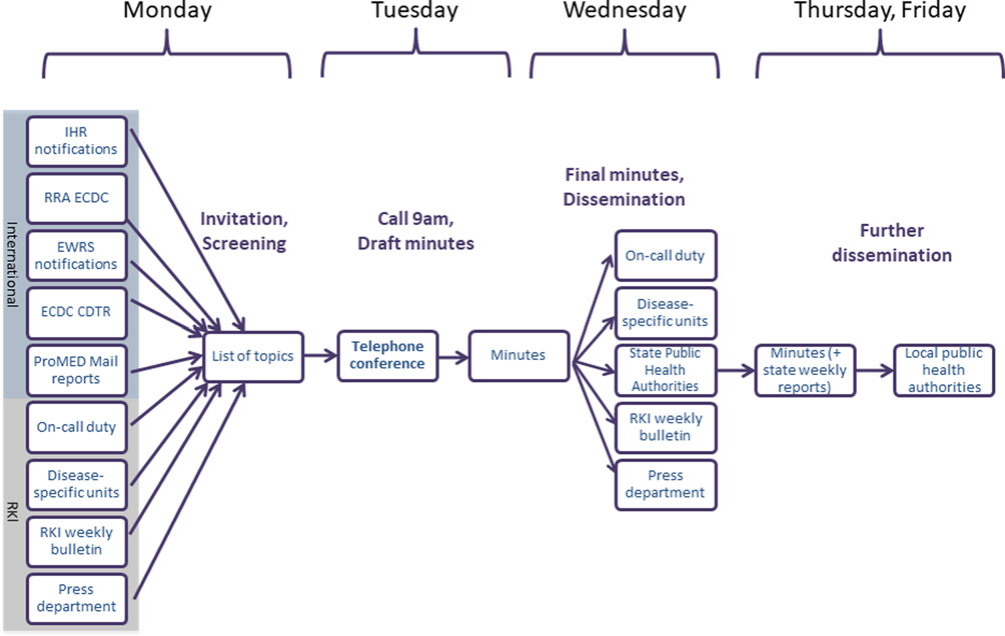
Overview of the weekly EpiLag procedures (at RKI), Germany, 2009–2018.

The EpiLag is organized by a team of RKI staff members. It includes an EpiLag coordinator, a weekly shifting editor and a moderator. The coordinator is a senior ID epidemiologist and responsible for the overall EpiLag concept: monitoring and evaluation, development and adaptation of Standard Operating Procedures (SOP), filling the staff roster, ensuring technical equipment and training and supporting the editors on duty. During an EpiLag duty week, the editor organizes the EpiLag following an SOP, which has been continuously improved and revised over the years. It includes step-by-step instructions for each day of the week as well as embedded hyperlinks for easy access to international sources and template e-mails. According to these, on Mondays, the editor screens selected international sources for relevant events, discusses the selected topics with subject matter experts in the disease-specific units and collects reports on national events and other topics. On Tuesday, they log the conference in writing and compile the EpiLag minutes using a standardized template. Presented reports on subnational events are provided by state PHA via email after the conference and embedded in the minutes. After an in-house editorial clearing process, the minutes are usually disseminated by email to the state PHA on Wednesday. The weekly EpiLag process is illustrated in detail in Figure 3 [4]. The moderator is the head of the unit. They lead through the conference on Tuesdays following a moderation scheme: first, they call every federal state to report presence and whether they have a topic to present; second, international and national events are presented by the editor on duty; third, subnational events are presented by representatives of the federal states, and finally everyone may bring up any other topic. Each topic is followed by a question and answer (Q&A) session.

## Monitoring and evaluation

The coordinator weekly monitors the conference in two Microsoft Excel spreadsheets. First, they collect weekly information on each federal state's attendance and on the duration of EpiLag. Secondly, they record each presented topic in a line list, documenting the presenter, the disease (category) discussed (e.g. salmonellosis, measles, …) and the respective week and year. So far, the EpiLag has been evaluated three times (in 2009, 2014 and 2019) [4]. All evaluations, which had alike aims and methodology, showed similar results.

## Methods

### Descriptive analysis

#### Data source

We used data of the two monitoring spreadsheets between 2009 and 2018. Topics were categorized into ID events, software-related topics and ‘other topics’. ID events were further subcategorized into international, national or subnational events. Events presented for the first time were defined as ‘new events’ others as ‘event updates’.

#### Data analysis

A descriptive analysis of the data of both spreadsheets was conducted with Excel. We calculated the average participation number of state PHAs per week and the mean duration of EpiLag in minutes. Furthermore, the frequency of the disease categories of events (‘new events’ and ‘event updates’ together) was determined, overall, by level and on average by conference. The number (and percentage amongst all reports in this disease category) of event updates were mentioned, when more than 10 updates existed for a particular event. It was not possible to analyse the frequency and percentage of event updates by reporting level, since some events started globally and later affected Germany as a national or subnational event and vice versa. Since the focus of the present manuscript lies on ID events, no further analysis of software-related topics or ‘other topics’ was conducted.

A sample of ID events from EpiLag was selected to illustrate the variety of topics and reporting pathways in the conference. For this, we chose not only international events with relevance for Germany (or vice versa), but also events relevant for global eradication goals, unusual events and events with complex measures.

### Evaluation of EpiLag

The most recent evaluation was conducted between March and May 2019 with the aim to assess the quality and usefulness of the EpiLag as well as to identify challenges and possible suggestions for improvement. For this, two participant groups were surveyed:

First, each of the 16 state PHAs received the questionnaire via email and was asked to fill in one questionnaire per PHA. It was up to the respective PHA to decide who to involve in filling in the questionnaire at their level. The questionnaire contained 34 closed and open questions regarding logistics, contents, minutes and overall usefulness of the EpiLag. Different Likert scales were used for closed questions: a five-level Likert scale for questions regarding logistics, content and minutes (corresponding to the answers, ‘strongly disagree’, ‘disagree’, ‘neutral’, ‘agree’, ‘strongly agree’) and a three-level Likert scale for the question regarding usefulness (corresponding to the answers ‘always’, ‘sometimes’, ‘never’). In addition, participants were asked to indicate selection criteria they apply for reporting events in EpiLag. The questionnaire was collected anonymously on paper either during a physical meeting at RKI or through letter mail.

Secondly, we administered an electronic questionnaire among the participants from the RKI. Each unit of the Department of Infectious Disease Epidemiology as well as the office of the head of the department was asked to complete one questionnaire in a non-anonymous way. The questionnaire was sent by email to the heads of the unit/department and each unit could choose whether they wanted to fill in the questionnaire together or delegate it to one respective employee. They could either print the filled-in questionnaire and return it by in-house mail or via email. The RKI questionnaire contained 29 open and closed questions, which touched upon the same four areas as the state questionnaire. There were no major differences in terms of content between the RKI questionnaire and the one of the state PHA. However, less detailed options for response to the closed questions (‘yes’ or ‘no’) could be chosen.

Both questionnaires contained open questions to trigger suggestions for improvement.

The answers from both questionnaires were entered into an Excel spreadsheet. Closed questions were analysed descriptively by frequency of category selected by the RKI unit/state PHA. Open questions regarding suggestions for EpiLag were screened one by one and counted in case they were named more than once. The most frequently named suggestions are reported in the results.

## Results

Between 2009 and 2018, a total of 484 EpiLag were held (45–51 times per year). An average EpiLag lasted 25 minutes (range: 4–60). On average 15 of 16 state PHAs (range: 9–16) joined the teleconference each week.

### Topics

From 2009 to 2018, a total of 4107 reports (new reports and event updates) were presented. Of those, 2975 reports (72%) covered ID events, 836 (20%) ‘other topics’ and 296 (7%) software-related topics. Of 2975 ID events, 1419 (48%) were presented by RKI and 1556 (52%) by the state PHAs. Of those reported by RKI, 1153 (81%) dealt with international and 266 (19%) with national events. Most frequent reports were about measles (532; 18%), salmonellosis (237; 8%) and influenza (147; 5%). On average 8.5 reports (range: 2–24) were discussed during an EpiLag teleconference, of which 6.1 covered ID events (range: 0–22)

#### International events reported by RKI

Of 1153 international events, reports were most frequently about Ebola virus disease (EVD; 108; 9.4%), measles (98; 8.5%), poliomyelitis (83; 7.2%), influenza (68; 5.9%), avian influenza (58; 5.0%), Middle East respiratory syndrome (MERS; 53; 4.6%) and salmonellosis (51; 4.4%) (Table 1). A total of 543 (47%) were newly reported events; most frequently about measles (53; 9.8%), influenza (37; 6.8%) and poliomyelitis (33; 6.1%)

**Table 1.** Most frequent infectious diseases categories reported within the topic of infectious disease events in EpiLag (by level), 2009–2018, Germany

International (*N* = 1153)^a^			National (*N* = 266)^a^			Subnational (*N* = 1556)^a^		
Disease	*n*	%	Disease	*n*	%	Disease	*n*	%
Ebola virus disease	108	9.4	Salmonellosis	58	21.8	Measles	402	25.8
Measles	98	8.5	Measles	32	12.0	Salmonellosis	128	8.2
Poliomyelitis	83	7.2	Influenza	22	8.3	Legionnaires’ disease	71	4.6
Influenza	68	5.9	Listeriosis	16	6.0	Norovirus gastroenteritis	70	4.5
Avian influenza	58	5.0	Haemolytic uremic syndrome	15	5.6	Hepatitis A	68	4.4
Middle East respiratory syndrome	53	4.6	Meningococcal meningitis	9	3.4	Influenza	57	3.7
Salmonellosis	51	4.4	Tuberculosis	8	3.0	Meningococcal meningitis	51	3.3
West Nile virus disease	39	3.4	Norovirus gastroenteritis	8	3.0	Unspecified gastroenteritis	46	3.0
Hepatitis A	37	3.2	Hepatitis A	7	2.6	Haemolytic uremic syndrome	45	2.9
Legionnaires’ disease	36	3.1	Unspecified gastroenteritis	6	2.3	Q fever	31	2.0
Zika virus disease	33	2.9	Adenovirus Keratoconjunctivitis	6	2.3	Shigellosis	28	1.8
Anthrax	32	2.8	Ebola virus disease	5	1.9	Tuberculosis	26	1.7
Dengue fever	32	2.8	Borna virus disease	5	1.9	Diphtheria	26	1.7
Chikungunya fever	28	2.4	Avian Influenza	4	1.5	Listeriosis	25	1.6
Yellow fever	23	2.0	Rabies	4	1.5	Botulism	24	1.5
Cholera	21	1.8	Q Fever	4	1.5	Typhoid fever	23	1.5
Crimean-Congo haemorrhagic fever	15	1.3	Vibrio infections	3	1.1	*Campylobacter* enteritis	21	1.4
Malaria	15	1.3	Hepatitis E	3	1.1	Vibrio infections	18	1.2
Meningococcal meningitis	15	1.3	West Nile virus disease	3	1.1	Cholera	18	1.2
Botulism	15	1.3	Hantavirus disease	3	1.1	Chickenpox	18	1.2
			Paratyphoid fever	3	1.1			

a Frequencies constitute overall frequencies, not stratified by ‘new event’ and ‘event update’.

#### National events reported by RKI

Of 266 national events, most frequent reports were about salmonellosis (58; 21.8%), measles (32; 12.0%), influenza (22; 8.3%), listeriosis (16; 6.0%) and haemolytic uremic syndrome (HUS; 15; 5.6%) (Table 1). A total of 141 (53%) were newly reported events; most frequently about measles (19; 7.1%), salmonellosis (19; 7.1%) and influenza (13; 4.9%).

#### Events reported by the state PHA

Of 1556 events reported by the state PHA, most frequent reports were about measles (402; 25.8%), salmonellosis (128; 8.2%), Legionnaires’ disease (71; 4.6%), norovirus gastroenteritis (70; 4.5%) and hepatitis A (68; 4.4%) (Table 1). A total of 1157 (74%) were new events; most frequently about measles (273; 23.6%), salmonellosis (84; 8.1%).

#### Event updates

Most event updates 63 (50% of all EVD reports in EpiLag) concerned the international epidemiological situation of the EVD outbreak in West Africa in 2013–2016. Among 63 MERS reports, 35 (56%) were updates, first ones from Saudi Arabia, later ones ‘worldwide’ reports. Avian influenza in 2009 was depicted in 28 event updates (43% of 65 total avian influenza reports). Among all 86 reports on poliomyelitis, 23 (27%) described the worldwide polio situation after the resurgence of cases in Syria in October 2013. Of in total 43 reports on anthrax, 22 (51%) and 8 (19%) reports, respectively, updated on outbreaks amongst injecting drug users in the United Kingdom and Europe in 2010 and 2012 [5]. The newly occurring Zika virus disease in the Americas in 2014 resulted in 41 reports in total over the last 10 years of EpiLag, of which 21 (51%) updated regularly on the situation worldwide. Among 532 reports on measles in EpiLag, most event updates regarded a series of 21 (4% of the total) and 9 (2%) event updates describing federal-state wide measles outbreaks in Hamburg in 2009 [6] and in Berlin in 2015 [7]. The pandemic influenza in 2009 had two threads of 15 event updates each (10% of all 147 influenza reports) in EpiLag and one thread of 14 event updates (10%) depicting the pandemic in different German federal states and worldwide. A series of 12 event updates was inside the category ‘HUS’ (18% of all 65 HUS reports) informing on a STEC/HUS outbreak originating in Northern Germany in 2011 [8, 9].

### Examples illustrating the variety of discussed topics (chronological order)

#### Cowpox in humans in Germany, 2009

At the second ever held EpiLag on 20 January 2009, human infections with cowpox were reported from different federal states. Cowpox is not classified as a notifiable disease in Germany. In the course of the upcoming weeks, information about the potential source of infections (rats) and likely associations to neighbouring countries were exchanged [10]. The event was disseminated internationally through EWRS. EpiLag helped gather information from the different federal states and to discuss the obligations and ways of notification and awareness raising [11].

#### STEC/HUS outbreak in Germany, 2011

From May to July 2011, a massive outbreak of gastroenteritis and HUS cases occurred mainly in the Northern part of Germany, caused by STEC of serotype O104:H4. Illnesses were associated with the consumption of sprouts [8]. In total, 3816 cases, including 845 cases of HUS and 54 deaths, were attributed to the outbreak [9]. Overall, 15 other countries were affected through travellers returning from Northern Germany. During a span of 10 weeks, the outbreak was discussed every week in the EpiLag mainly regarding the epidemiological situation, specific clusters, pathogen characteristics, laboratory investigations and sample transport, public health measures (e.g. exclusion criteria for visiting community facilities or re-admission to work), epidemiological studies and preparedness and response (P&R) activities.

#### EVD in West Africa, 2013–2016

From December 2013 to September 2016, the largest ever recorded outbreak of EVD took place (mainly) in West Africa [12]. Germany was only affected indirectly, by treating three EVD patients (evacuated from West Africa) and through the quarantine of a person with a needle stick injury (who did not develop EVD) [13–16]. During the roughly 2 years of the outbreak, it was discussed in EpiLag 63 times mainly regarding the epidemiological situation, travel-related cases and P&R activities in Germany.

#### Botulism associated with the consumption of fish, 2016

During the EpiLag on 15 November 2016, a state PHA reported a case of laboratory-confirmed botulism potentially associated with the consumption of vacuum-packed fish. This triggered another state PHA to describe a suspected case of botulism in their federal state, where also a fish product was the suspected source of infection. In both patients, botulinum neurotoxin E had was diagnosed a few weeks later. RKI informed other European countries through EPIS and EWRS in November 2016. Until the end of 2016 a total of six foodborne botulism cases associated with the consumption of dried and salted roach [17] were reported in Germany and Spain. Main discussion points over eight calendar weeks in which this event was discussed in the EpiLag were the epidemiological situation, laboratory results, the source of infection and public health measures (e.g. trace back results from the food safety authorities).

#### Measles contact tracing, 2017

In 2017, several state PHAs reported difficulties (e.g. how to receive passenger manifests and data protection issues) with contact tracing for infectious measles cases on aircraft in EpiLag. These triggered discussions were deferred to one of the regular physical meetings and led to published results [18].

#### Evaluation of EpiLag

**Fig. 4.**
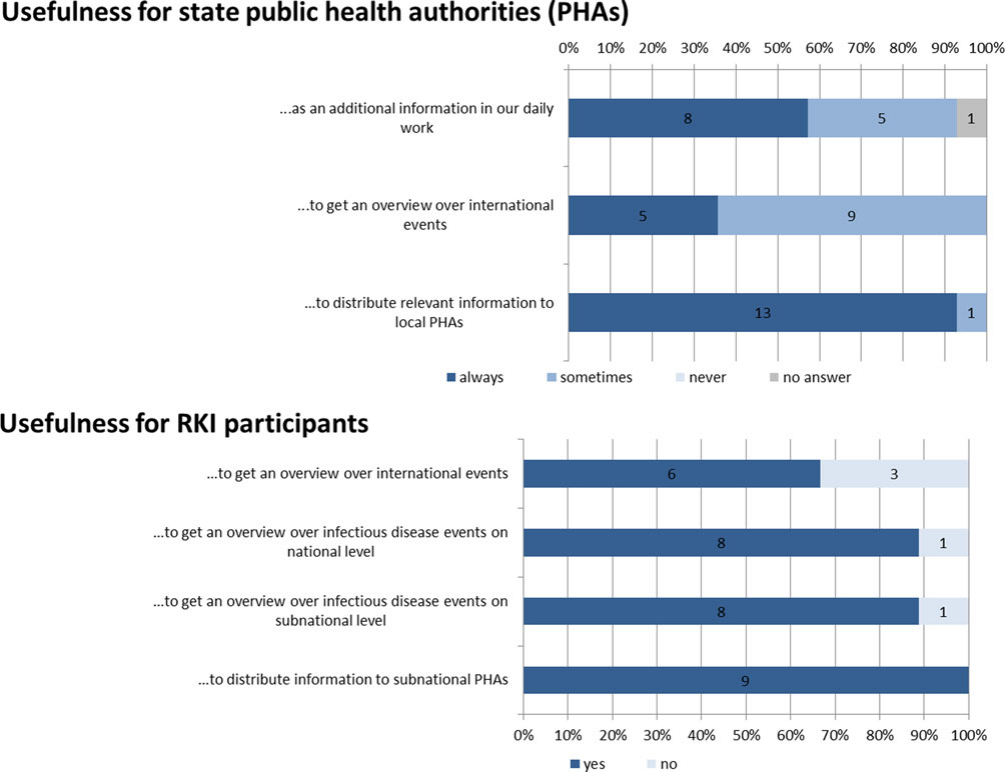
Answers assessing the usefulness of the EpiLag for state public health authorities and RKI participants, Germany, 2009–2018.

Representatives of 14/16 (88%) state PHAs responded to the questionnaire. All 14 ‘agreed’ or ‘strongly agreed’ that the overall logistics of the EpiLag (time, duration and timeframe for presenting reports) and the selection of participants (restricted to national and state PHA) are appropriate, and that the organization is reliable. Between ten and 14 ‘agreed’/‘strongly agreed’ that the minutes are timely (*n* = 14), clear (*n* = 14), correct (*n* = 13 for the ID event reports and *n* = 10 for the Q&A portion) and appropriate in terms of length (*n* = 13). The vast majority of state PHAs (*n* = 13) ‘agreed’/‘strongly agreed’ that the contents reported by RKI during the EpiLag are appropriate. International topics are seen as informative (*n* = 14 ‘agreed’/‘strongly agreed’) and relevant for the daily work (*n* = 11 ‘agreed’/‘strongly agreed’, *n* = 2 chose ‘neutral’ and *n* = 1 ‘disagreed’). Topics presented by other state PHA were deemed appropriate by 13 state PHAs (‘neutral’: *n* = 1). Ten and 12 out of 14 state PHAs, respectively, ‘agreed’/‘strongly agreed’ that the structure and presented topics of the EpiLag stimulate discussion and encourage a low-threshold exchange of information. All state PHAs for which this information was available (*n* = 7) agreed that EpiLag should continue to enable ‘off-the-record’ information and reports. Eight state PHAs (all for which this information was available) use the content of the EpiLag ‘always’ and five PHAs ‘sometimes’ as a source of information for their daily work. Five and nine, respectively, also to ‘always’ and ‘sometimes’ get an overview over international events. The information exchanged is rarely kept for internal use only (‘always’: *n* = 4) and 13 state PHAs disseminate the minutes ‘always’ to local PHA (Fig. 4). The majority of state PHA (*n* = 11) passes them on further to political stakeholders (not shown in Fig. 4). It was suggested by the PHA that RKI could increase the reporting on relevant challenges and new developments at the institute and to upscale EpiLag on demand in times of crisis.

Many state PHAs (*n* = 6) specified to select an event for reporting if it affects or shows relevance to ⩾1 federal state, when they assess the event as highly unusual or rare (e.g. transmission route, people affected, pathogen; *n* = 8) or when it includes a high percentage of severe infections (*n* = 1). Selected PHAs also choose events, because they encounter problems in their management or reporting and deem it useful to exchange experiences or best-practices with other PHA.

Representatives of all eight units of the Department for Infectious Disease Epidemiology at RKI and one representative of the department's head's office partook in the evaluation (nine respondents). All nine RKI respondents confirmed that they ensure participation of minimum one person of their organizational unit in EpiLag and that the circle of participants for EpiLag is appropriate. All rated the minutes as clear and appropriate in length and found that their comments in the feedback loop were adequately implemented into the minutes. All seven RKI respondents (for which data was available for this question) agreed that the EpiLag fosters an open and low-threshold information exchange. Regarding the content, five and eight units agree that the international and national topics are informative, respectively. All RKI participants regard the EpiLag as useful for disseminating information and recommendations to state and local PHA (*n* = 9 selected ‘yes’), most appreciate it to get an overview about events at national, subnational (‘yes’ by *n* = 8 each) and international level (‘yes’ by *n* = 6) (see Fig. 4).

For suggestions some RKI participants advocated for federal state PHAs to report with an even lower threshold and to focus international reports on events in neighbouring countries and countries directly related to Germany through travel.

Furthermore, it is known from personal conversation with federal state participants that the EpiLag was also perceived to foster the exchange and network of German surveillance stakeholders.

## Discussion

The EpiLag has been established for 10 years as a telephone conference with voluntary participation of ID epidemiologists from federal state level. The continuously high participation demonstrates its acceptance and importance. Factors contributing to this success are the relevance of presented topics, organisation (based on SOPs) and the possibility for discussion. EpiLag's well-structured format and moderation scheme allows a substantially high number of topics to be presented in a short time period (time efficiency) and to keep participants engaged during well-balanced discussion parts. Topics that could trespass the intended time frame are postponed to the face-to-face meetings, unless very urgent.

The EpiLag format allows to bring in ID events, software-related issues and ‘other topics’. Those are especially important to keep the mandatory surveillance system running.

The regular screening of international sources for ID events with relevance for Germany serves as an event-based surveillance tool and helped to pool epidemic intelligence activities within the RKI [19].

International events seem of interest to most participants, although the relevance for daily work is highest for events in neighbouring countries or countries with a link to Germany through travel. However, as international ID events presented during the EpiLag from 2009 to 2018 comprise the four Public Health Emergencies of International Concern (PHEICs) declared within this time period (pandemic influenza [20], poliomyelitis [21], EVD in West Africa [12] and Zika virus disease [22]) it is proven necessary to continue informing the subnational levels as soon as possible on events, which are maybe outside the most relevant geographical areas, but might potentially pose a threat to public health worldwide and cause increased work load or attention in Germany. Another recent example of this kind, the early and effective information of the subnational levels in January 2020 on an outbreak of unknown pneumonias in China – later to be declared a PHEIC as coronavirus disease 2019 (COVID-19) – justifies keeping our selection criteria used for international events as sensitive as possible.

The EpiLag allows the RKI to alert subnational PHAs of (potential) multi-federal-state outbreaks, which may have been detected through molecular typing and to convey recommendations, for example, for PHAs to link existing cases to these outbreaks, interview cases regarding a common suspected source or initiate molecular analysis (for potential linking of cases) [23].

An event with a high number of updates at national level was the STEC/HUS-outbreak in Germany (2011) [9]. This can be explained by the extent of the outbreak over multiple federal states, and by the outbreak investigation being coordinated through national level crisis management structures at RKI. In this context (as well as during the EVD outbreak in West Africa) the EpiLag was used to support the harmonisation of control measures between federal states by discussing options for P&R activities.

Although never officially discussed or agreed upon in 10 years, the fact that state PHA seem to apply similar selection criteria for reporting events shows that the EpiLag is perceived as useful for similar matters throughout all federal states. Hence, mainly events with the possibility to be detected at local level (e.g. point-source events of gastrointestinal infections, measles or respiratory diseases) account for nearly 50% of subnational level reports and function for sharing information on outbreak identifiers or sources/places of exposure with cross-federal-state significance.

Furthermore, state PHAs frequently use ID events presented at the EpiLag to exchange experiences regarding control measures: Contact tracing of individuals exposed to a case of ID on an aircraft is for example challenging in various aspects. Reports and discussions during the EpiLag and several face-to-face meetings resulted in a common recommendation on contact tracing for German PHA and a publication on this topic [18]. This example additionally highlights how the EpiLag helped to identify and discuss challenges and best practices within routine public health structures and how it has assisted in harmonising control measures between the federal states.

The described example of botulism cases associated with the consumption of fish in 2016 shows how EpiLag can facilitate the linkage of cases of two federal states events and (with the help of further epidemiological investigation and Europe-wide databases) the subsequent detection of case clusters of international significance [17].

Measles accounted for 17% of all reports and was considered to be of high importance in the international-, national- and state-level context. The reports varied from descriptions of community outbreaks [24, 25], activities at national level [26–28], distribution of ECDC RRA [28] to updates concerning the measles elimination activities of WHO [29]. Thereby the EpiLag constitutes a tool to support the process of measles elimination in Germany.

All EpiLag evaluations conducted so far showed similar results, with an overall positive feedback. Suggestions in relation to logistics and organization were used for continuously improving the SOP for EpiLag. Suggestions regarding content (e.g. the desired more sensitive reporting of state PHA's documents and events as well as of recent developments at RKI) were fed back to state PHA and the heads of units and the department of Infectious Disease Epidemiology at RKI. To ensure a low-threshold exchange and confidentiality of information inside the public health sector, it was not only important to keep the participant circle limited (as both participant groups wished), but also to continue ‘off-the-record’ reporting of ID events or information of any kind in EpiLag. This possibility was cherished highly by state PHA and could enable more sensitive reporting from their side.

Overall, the evaluation results support the assumption that both the subnational and national participants rate the EpiLag as an easy-to-use, effective and useful tool for low-threshold exchange of information. State PHA use the EpiLag minutes to raise awareness in their respective states about relevant ID events, which suggests that EpiLag may thereby also serve early warning functions.

With its existing format, a fixed weekly telephone conference, the EpiLag does not intend to substitute timely and bilateral communication between subnational and national level. The state PHAs suggestion to scale up the EpiLag during ID crises is being considered. In fact, a scale-up of EpiLag to be held twice a week was successfully applied during periods of the COVID-19 crisis in 2020 to ensure efficient timely exchange.

It is to note that EpiLag and also our analysis has several limitations. First, it is possible that although a disease category was reported as an ID event less frequently in EpiLag, this disease was discussed more frequently in terms of its surveillance and diagnostic strategy, case definitions or disease-specific software-entry mask under ‘other’ or software-related topics. A subclassification for ‘other’ topics and software-related topics regarding the disease they potentially referred to was not routinely introduced into the monitoring sheets until 2018 (but only later in 2021) and could therefore not be taken into consideration in this paper. This applies also to the introduction of an ‘ID event identifier’ in the monitoring sheet to clearly distinguish how many event updates refer to one particular outbreak.

Although we believe that the EpiLag has been used successfully to discuss a large number of events in the past 10 years, it needs to be noted that, due to voluntary participation and reporting of events, one limitation of EpiLag is that it (and therefore also our analysis) does not offer a complete picture of the occurrence of ID events in Germany.

## Conclusion

The EpiLag is a highly valued, useful and effective tool to support the communication and information exchange between the national, state and local PHA in Germany regarding ID events. With its routinely implemented procedures, the EpiLag not only serves its objectives, but also offers important support in times of crises. Through the fact that it has built trust and constitutes an added value by benefiting from each other's experience, it established a low threshold exchange. This further strengthens the network and interpersonal relationships between stakeholders in the surveillance system. We recommend the EpiLag as a simple and time-efficient tool for other countries, further sectors or for regular inter-sectoral communication.

As a result of this recommendation, the effective format of EpiLag has been recognized internationally in some of RKI's partner countries, such as Albania, Montenegro, North Macedonia, Kosovo and Tunisia. Since 2015, three of those countries successfully adapted the EpiLag to their own contexts and implemented it as a tool to foster horizontal exchange (between subnational entities) inside their centralized public health structures [30].

## Data Availability

The data that support the findings of this study are available from the authors.
